# Suboptimal community growth mediated through metabolite crossfeeding promotes species diversity in the gut microbiota

**DOI:** 10.1371/journal.pcbi.1006558

**Published:** 2018-10-30

**Authors:** Michael A. Henson, Poonam Phalak

**Affiliations:** 1 Department of Chemical Engineering, University of Massachusetts, Amherst, Massachusetts, USA; 2 Institute for Applied Life Sciences, University of Massachusetts, Amherst, Massachusetts, USA; Ecole Polytechnique Fédérale de Lausanne, SWITZERLAND

## Abstract

The gut microbiota represent a highly complex ecosystem comprised of approximately 1000 species that forms a mutualistic relationship with the human host. A critical attribute of the microbiota is high species diversity, which provides system robustness through overlapping and redundant metabolic capabilities. The gradual loss of bacterial diversity has been associated with a broad array of gut pathologies and diseases including malnutrition, obesity, diabetes and inflammatory bowel disease. We formulated an *in silico* community model of the gut microbiota by combining genome-scale metabolic reconstructions of 28 representative species to explore the relationship between species diversity and community growth. While the individual species offered a broad range of metabolic capabilities, communities optimized for maximal growth on simulated Western and high-fiber diets had low diversities and imbalances in short-chain fatty acid (SCFA) synthesis characterized by acetate overproduction. Community flux variability analysis performed with the 28-species model and a reduced 20-species model suggested that enhanced species diversity and more balanced SCFA production were achievable at suboptimal growth rates. We developed a simple method for constraining species abundances to sample the growth-diversity tradeoff and used the 20-species model to show that tradeoff curves for Western and high-fiber diets resembled Pareto-optimal surfaces. Compared to maximal growth solutions, suboptimal growth solutions were characterized by higher species diversity, more balanced SCFA synthesis and lower exchange rates of crossfed metabolites between more species. We hypothesized that modulation of crossfeeding relationships through host-microbiota interactions could be an important means for maintaining species diversity and suggest that community metabolic modeling approaches that allow multiobjective optimization of growth and diversity are needed for more realistic simulation of complex communities.

## Author summary

The gut microbiota serve a critical role in maintaining a healthy state in the human host. The gut contains approximately 1,000 bacterial species that provide a wide range of metabolic capabilities including the breakdown of dietary compounds and the synthesis of useful metabolites. The robust function of the gut community is intimately connected to its diversity, both with respect to the number of species and the relative abundance of those species. The gradual loss of diversity is a key element of microbiota dysbiosis, which has been correlated with a wide range of health problems including inflammatory bowel disease. To investigate species diversity in the gut microbiota, we developed an in silico community model by combining genome-scale metabolic reconstructions of 28 representative species from the most abundant genera in the human gut. Our model predicted that maximal community growth produced low species diversity and no synthesis of the health-promoting metabolite butyrate. After reducing the community model to 20 species, we showed that suboptimal community growth allowed much higher species diversity and butyrate synthesis more consistent with in vivo studies. The model predicted that increased diversity could be achieved through modulation of metabolite crossfeeding relationships between species, an experimentally testable hypothesis.

## Introduction

The gut microbiota comprise a highly complex ecosystem that has been characterized as an additional organ within the human host [1, 2]. The microbiota form a mutualistic relationship with the host, with saccharolytic species enzymatically degrading complex carbohydrates into fermentable sugars and fermentative species converting sugars and other available nutrients into a variety of absorbable metabolites [3, 4]. A particularly important function of the microbiota is to ferment dietary fiber into the short-chain fatty acids (SCFAs) acetate, butyrate and propionate [5, 6]. While significant variations are possible depending on diet, the molar ratio of these three SCFAs is approximately 60:20:20 [7]. SCFAs are consumed by host colonocytes as a primary energy source, with butyrate being the preferred SCFA but acetate probably supplying more energy to its higher concentration *in vivo*.

The gut microbiota consist of approximately 1,000 species [8] and 7,000 unique strains [2] in a typical human host. The two dominant phyla in healthy humans are Firmicutes and Bacteroidetes, which comprise more than 90% of the community [9, 10]. Other important but much less abundant phyla are Proteobacteria, Actinobacteria, Euryarchaeota and Verrucomicrobia as well as Eukaryota such as fungi [11, 12]. Metagenomic studies have shown wide variations in bacterial composition in healthy humans [13, 14], demonstrating that microbiota composition is an individual characteristic and an inadequate measure for assessing gut health across patient populations.

A hallmark of healthy gut communities is high diversity [15, 16], both in terms of the species present and the relative abundance of these species [17, 18]. An integrated gene catalog developed from 1,267 sequenced samples and comprising almost 10 million genes provides detailed information on the number of genes from over 700 gut genera [19]. The high diversity of the gut microbiota is demonstrated by 75 genera having relative gene counts (genes counts in that genus divided by total gene counts) of at least 0.1%. Numerous studies have shown a strong correlation between bacterial diversity and health/disease states, with long-term loss of diversity a key characteristic of dysbiosis [20–22]. The loss of bacterial diversity has been implicated in broad range of diseases including *Clostridium difficile* infections [23], inflammatory bowel and Crohn’s diseases [24, 25], obesity [26], diabetes [27], cardiovascular disease [28], rheumatoid arthritis [29], colorectal cancer [30], cystic fibrosis [31] and depression [32].

Genome-scale metabolic modeling has emerged as an important tool for computationally interrogating the metabolism of microbial communities. A number of alternative methods for combining metabolic reconstructions of single species into community models are now available. These methods are invariably based on growth rate maximization, either with regard to the species individually [33, 34] or the community as a whole [35–37]. Compared to other techniques, the recently developed SteadyCom method represents an important advance by performing community flux balance analysis (FBA) to determine the relative abundance of each species for maximal community growth while ensuring that all metabolites are properly balanced within each species and the community [38]. While the usual FBA objective of maximal growth has been experimentally demonstrated for individual bacteria such as *Escherichia coli* [39, 40], limited data is available to support the adoption of maximal growth as a community objective [41–43]. Indeed this objective has the potential to favor the fastest growing species and produce communities with low diversity that are inconsistent with healthy gut communities observed *in vivo*. In this study, we utilized the SteadyCom method to investigate the tradeoff between community growth and species diversity for *in silico* communities comprised of 20 and 28 representative gut species.

## Materials and methods

### Gut microbiota models

Semi-curated genome-scale metabolic reconstructions for representative species within the 28 most abundant genera [19] in the human gut were obtained from the Virtual Metabolic Human database (vmh.uni.lu) [44]. These models represented the five major bacterial phyla (Actinobacteria, 2 species; Bacteroidetes, 4 species; Firmicutes, 15 species; Fusobacteria, 1 species; Proteobacteria, 6 species), including 10 species from the highly prevalent Firmicutes order Clostridia (Table 1). The 28 genera covered almost 85% of reference genes by occurrence frequency according to a recent integrated catalog of the human gut microbiota [19]. Each species was constrained according to either a Western or high-fiber diet [44] and assigned a non-growth ATP maintenance (ATPM) value of 10 mmol/gDW/h, which is within the range reported for curated bacterial reconstructions. Because the species biomass equations were not curated [44], all species models used the same growth-dependent maintenance energy. The community metabolic model was constructed from the single-species reconstructions using the createCommModel function provided within the SteadyCom suite of MATLAB tools [38]. The community model accounted for 22,203 genes, 26,867 metabolites and 35,031 reactions within the 28 species as well as 354 uptake and 354 secretion reactions for the extracellular space.

**Table 1:** Properties of the 28 genome-scale metabolic reconstructions comprising the gut microbiota model.

Number^+^	Species*	Genes	Metabolites	Reactions
1/1	*Bacteroides thetaiotaomicron*	841	1032	1370
2/2	*Clostridium clostridioforme*	1277	990	1352
3/3	*Faecalibacterium prausnitzii*	619	825	1008
4/4	*Eubacterium rectale*	700	941	1194
5/5	*Blautia wexlerae*	854	1006	1248
6/6	*Streptococcus salivarius*	604	868	1156
7/7	*Ruminococcus callidus*	564	912	1138
8/8	*Collinsella tanakaei*	691	959	1239
9/9	*Escherichia coli*	1193	1152	1795
10/10	*Roseburia inulinivorans*	663	903	1097
11/-	*Lactobacillus mucosae*	582	852	1080
12/11	*Prevotella ruminicola*	695	916	1119
13/-	*Alistipes finegoldii*	667	882	1139
14/-	*Bifidobacterium longum*	529	729	890
15/12	*Enterobacter cloacae*	1330	1223	1776
16/13	*Klebsiella pneumoniae*	1395	1186	1801
17/14	*Coprococcus catus*	824	969	1211
18/15	*Veillonella atypica*	663	895	1120
19/-	*Parabacteroides distasonis*	797	1049	1357
20/16	*Fusobacterium varium*	805	963	1228
21/17	*Haemophilus parainfluenzae*	656	973	1257
22/-	*Pseudoflavonifractor capillosus*	678	885	1070
23/18	*Dorea longicatena*	689	851	1017
24/19	*Citrobacter amalonaticus*	1317	1218	1811
25/-	*Phascolarctobacterium succinatutens*	560	832	1018
26/20	*Desulfovibrio desulfuricans*	717	1022	1279
27/-	*Megasphaera elsdenii*	649	931	1172
28/-	*Acidaminococcus fermentans*	644	903	1089
	**Total 28 species model**	**22203**	**26867**	**35031**
	**Total 20 species model**	**17097**	**19804**	**26216**

*underlined species are not included in the 20 species community

^+^species numbers for 28 species/20 species communities

The uptake reactions in the extracellular space of the community model were constrained with the chosen diet as the union of all the maximum nutrient uptake rate constraints from the 28 species models. An Excel file with community uptake constraints for the four models (20 and 28 species; Western and high-fiber diets) is available in the Supporting Information (S1 File). With the exceptions noted below, the maximum nutrient uptake rates of each species were set equal to values defined by the chosen diet for that species. This approach ensured that each species would produce the same single-species growth rate with SteadyCom as obtained with standard flux balance analysis (FBA). Crossfeeding of all 21 amino acids and eight common metabolic byproducts (acetate, CO_2_, ethanol, formate, H_2_, D-lactate, L-lactate, succinate) was promoted by increasing the maximum nutrient uptake rates of these nutrients to 10 mmol/gDW/h. A single value was used due to the lack of metabolite- and species-dependent data for byproduct uptakes in the literature. While these constraints had no effect on single-species metabolism due to the extracellular constraints, they allowed species with different single-species growth rates to coexist through crossfeeding. The SCFAs butyrate and propionate were not allowed to be consumed under the assumption that crossfeeding of these two SCFAs was negligible compared to utilization by the host [45].

### Community simulations

The community metabolic models were solved with the SteadyComCplex function within SteadyCom [38]. The IBM ILOG Cplex solver was used for linear program (LP) solution. SteadyCom performs community FBA by computing the relative abundance of each species for maximal community growth while ensuring that all metabolites are properly balanced within each species and the community. SteadyCom provides the capability to constrain the species abundances to explore various features of community behavior. Most simulations were performed with the default abundance constraints (lower bound zero, upper bound unity), which is referred to as the unconstrained case. Community flux variability analysis (FVA) was performed with respect to the species abundances using the SteadyComFVACplex function within SteadyCom. FVA showed that all maximal growth communities had unique species abundances. Using community FVA results for the 28-species community, species which could only coexist at 70% or less of the maximal community growth rate were eliminated to yield a 20-species community (Table 1). The 20-species model was used to explore the tradeoffs between community growth and diversity by constraining the species abundances with upper bounds computed from community FVA results obtained at different percentages of the maximal growth rate (see below).

Simulation results were analyzed with respect to the community growth rate, species abundances and diversity in the community, and the number and type of crossfeeding relationships. The growth rate and species abundances were direct outputs of SteadyCom. Species diversity was quantified using the Inverse Simpson equitability index [46–48] that accounted both for the number of participating species (e.g. richness) and the abundance of each species (e.g. evenness),

$$\begin{array}{c} D_{com}=\frac{1}{N}\frac{1}{\sum_{i=1}^{N}p_{i}^{2}} \end{array}$$ (1)

where *N* is the total number of species and *p*_*i*_ ∈ [0, 1] is the relative abundance of species *i*, another direct output of SteadyCom. The diversity measure *D*_*com*_ varied from $\frac{1}{N}$ if the community has a single participating member to unity if the all species participated and had the equal abundances. While the community models were built from metabolic reconstructions of particular species, the species name and associated genus were used interchangeably since the modeling goal was to achieve diversity in the genera.

To investigate the tradeoff between community growth and species diversity, maximum abundances in the 20-species community model were constrained using FVA results as follows,

$$\begin{array}{c} p_{j}^{U}=2r\frac{p_{j}^{max}}{\sum_{i=1}^{N}p_{i}^{max}} \end{array}$$ (2)

where $p_{j}^{max}$ is the maximum abundance of species *j* calculated at a specified percentage of the maximal growth rate using FVA, *r* is a uniform random number in the closed interval [0 1] and $p_{j}^{U}$ is the upper abundance bound of species *j*. The maximum abundance of species *j* was divided by the sum of the maximum abundances of all species such that the scaled maximum abundances summed to unity. The bounds were randomized to more completely sample the growth-diversity tradeoff and were generated subject to the constraint $\sum_{j=1}^{N}p_{j}^{U}\geq 1$ to avoid producing structurally infeasible LP problems. The multiplier 2 was introduced because *r* was uniformly distributed with expected value 0.5. As compared to completely random bounds, Eq (2) allowed species with higher FVA abundances to have higher upper bounds on average. These bounds tended to constrain the solution such that higher diversity than the optimal solution was obtained, especially when FVA solutions at lower growth rates (*e.g.* 60% of maximal) were used. Eq (2) is heuristic in the sense that the calculated upper bounds do not ensure Pareto optimality [49, 50] of the resulting solutions in the growth-diversity space. More sophisticated methods that allow the calculation of the maximal diversity at a given growth rate would be required for this purpose. For each diet, a total of 900 cases were performed at FVA growth rates 60–99% of the maximal value, with more cases run at lower growth rates (150 cases at 60% and 65%, 125 cases at 70% and 75%, 100 cases at 80%, 85% and 90%, 25 cases at 95% and 99%) to adequately sample higher diversities. When $\sum_{j=1}^{N}p_{j}^{U}$ was close to unity, SteadyCom often returned a solution where $\sum_{i=1}^{N}p_{i}^{max}$ was outside the default tolerance of 10^−4^ specified within SteadyCom. These solutions were discarded to maintain the same accuracy of all suboptimal growth solution, substantially reducing the total number of cases (*e.g.* 900 to 595 for the Western diet).

## Results

### Single species provide a broad range of metabolic capabilities

**Fig 1:**
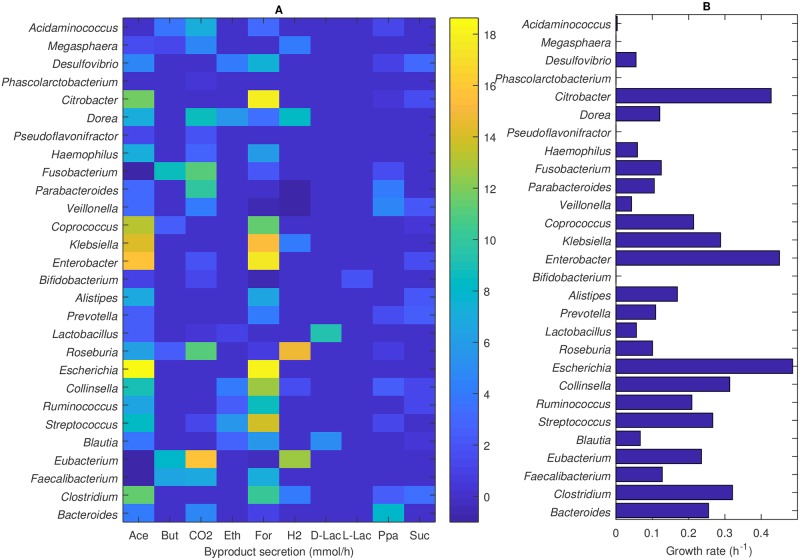
(A) Byproduct synthesis rates and (B) growth rates of the 28 species on an *in silico* Western diet. The species are listed by their genera. The byproducts listed are acetate (ace), butyrate (but), carbon dioxide (CO_2_), ethanol (eth), formate (for), hydrogen (H_2_), D-lactate (D-lac), L-lactate (L-lac). propionate (ppa) and succinate (suc).

First the community model was constrained to investigate the metabolic capabilities of each species individually on the *in silico* Western diet. These single-species simulations were performed within SteadyCom by constraining the abundance of all other species to zero. The growth rate and the secretion rates of ten primary metabolic byproducts were determined for each species (Fig 1). The 28 species exhibited a wide range of growth rates, including three species (*Escherichia*, *Enterobacter*, *Citrobacter*) with growth rates exceeding 0.4 h^−1^ and four species (*Bifidobacterium*, *Pseudoflavonifractor*, *Phascolarctobacterium*, *Megasphaera*) with growth rates of zero for the ATP maintenance value of 10 mmol/gDW/h. While species with high individual growth rates were expected to have a competitive advantage in the *in silico* community, slower growing species had the possibility of coexisting by increasing their growth rates through metabolite crossfeeding. With regard to SCFA synthesis, 24 species secreted acetate, 7 species secreted butyrate including 3 major butyrate producers (*Faecalibacterium*, *Eubacterium*, *Fusobacterium*), and 13 species secreted propionate including 3 major propionate producers (*Bacteroides*, *Veillonella*, *Parabacteroides*). While the SCFA synthesis capabilities of the genera *Bacteroides*, *Faecalibacterium* and *Eubacterium* are well documented [51, 52], the other SCFA predictions also appear to be consistent with experimental studies [53, 54].

### Optimal community growth leads to low species diversity

**Fig 2:**
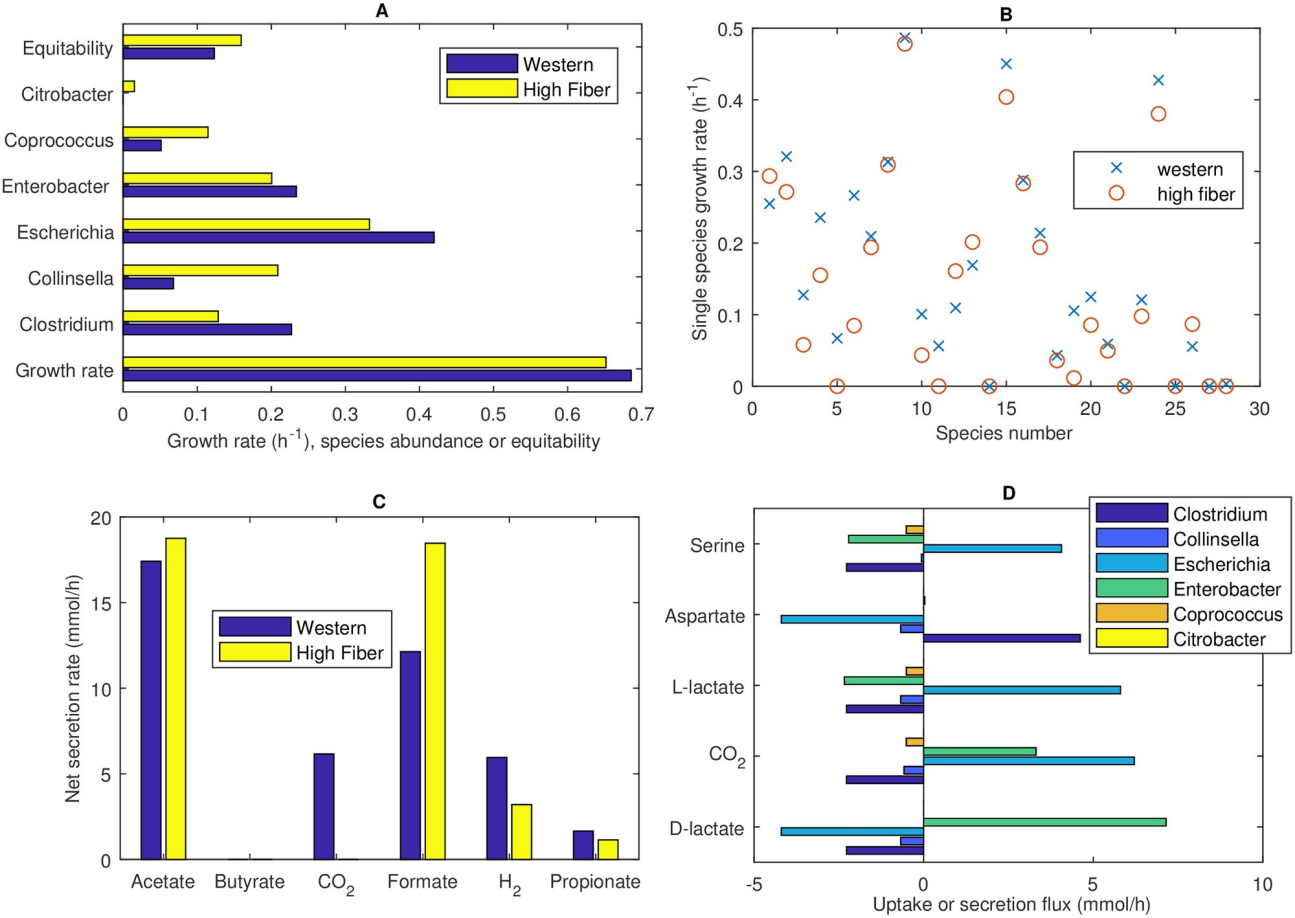
Optimized 28-species communities for *in silico* Western and high-fiber diets. (A) Growth rate (h^−1^), non-zero species abundances and equitability measure *D*_*com*_. (B) Single-species growth rates (h^−1^) with species numbers corresponding to Table 1. (C) Net synthesis rates of major metabolic byproducts including the three SCFAs acetate, butyrate and propionate. The byproducts ethanol, D-lactate, L-lactate and succinate are not shown because their net secretion rates were zero. (D) Uptake and secretion fluxes of the five metabolites most significantly crossfed between the participating species for the Western diet.

SteadyCom was used to determine the optimal growth rate and species abundances of the 28-member community on Western and high-fiber diets. The growth rate on each diet was similar (0.69 h^−1^ Western diet, 0.65 h^−1^ high-fiber diet) (Fig 2A) and appeared to be consistent with limited data available for *in vivo* gut community growth rates [55, 56]. Each community consisted of a small number of species, with only five species for the Western diet and six species for the high-fiber diet having non-zero abundances. The abundances of the 28 species correlated strongly with their single-species growth rates (Fig 2B; *P* < 10^−4^ for either diet), as would be expected from a community modeling methodology based on growth maximization. The dominant species included generally beneficial commensals from the genera *Clostridium* [57], *Collinsella* [58] and *Coprococcus* [59] but also represented several genera associated with inflammatory bowel disease (IBD) pathogenesis, including *Escherichia* [60], *Enterobacter* [61] and *Citrobacter* [62]. As a result of dominance by a few species, both communities exhibited low diversity, which has been correlated with a wide variety of gut pathologies [20–22]. Only slightly higher diversity was achieved for the Western diet when the ATP maintenance value of each species was tuned to the extent possible to achieve a uniform single-species growth rate of 0.2 h^−1^ (S1 Fig), demonstrating that the domination of particular species was partially attributable to their ability to more effectively exploit crossfed metabolites for growth.

The community model was formulated to allow crossfeeding of all 21 amino acids and eight metabolic byproducts. Ethanol, D-lactate, L-lactate and succinate were crossfed to the extent that their net secretion rates (difference between the sum of the species synthesis and uptake rates) on either diet were zero (not shown). Both diets produced relatively high formate and acetate net secretion rates (Fig 2C), while the rates of CO_2_, H_2_, propionate were comparatively low and no butyrate was produced. Predicted ratios of the acetate:butyrate:propionate rates of 91:0:9 and 94:0:6 for the Western and high-fiber diets, respectively, were inconsistent with reported *in vivo* SFCA levels, which commonly are in the range of 60:20:20 [7]. The SCFA imbalance predicted for both diets was attributable to acetate production by all participating species, a lack of propionate producers (only *Clostridium* and *Collinsella*), and the absence of acetate consumers and butyrate producers. The five most significantly cross-fed metabolites were predicted to be the amino acids aspartate and serine and the byproducts D-lactate, L-lactate and CO_2_ (Fig 2D). *Clostridium*, *Escherichia* and *Enterobacter* formed a mutualistic three-species subcommunity with both large uptake and secretion rates of the five metabolites. By contrast, *Collinsella* and *Coprococcus* exhibited commensal interactions by solely consuming the secretion products while *Citrobacter* did not participate in the crossfeeding of these metabolites.

### Suboptimal community growth offers the potential for enhanced species diversity

**Fig 3:**
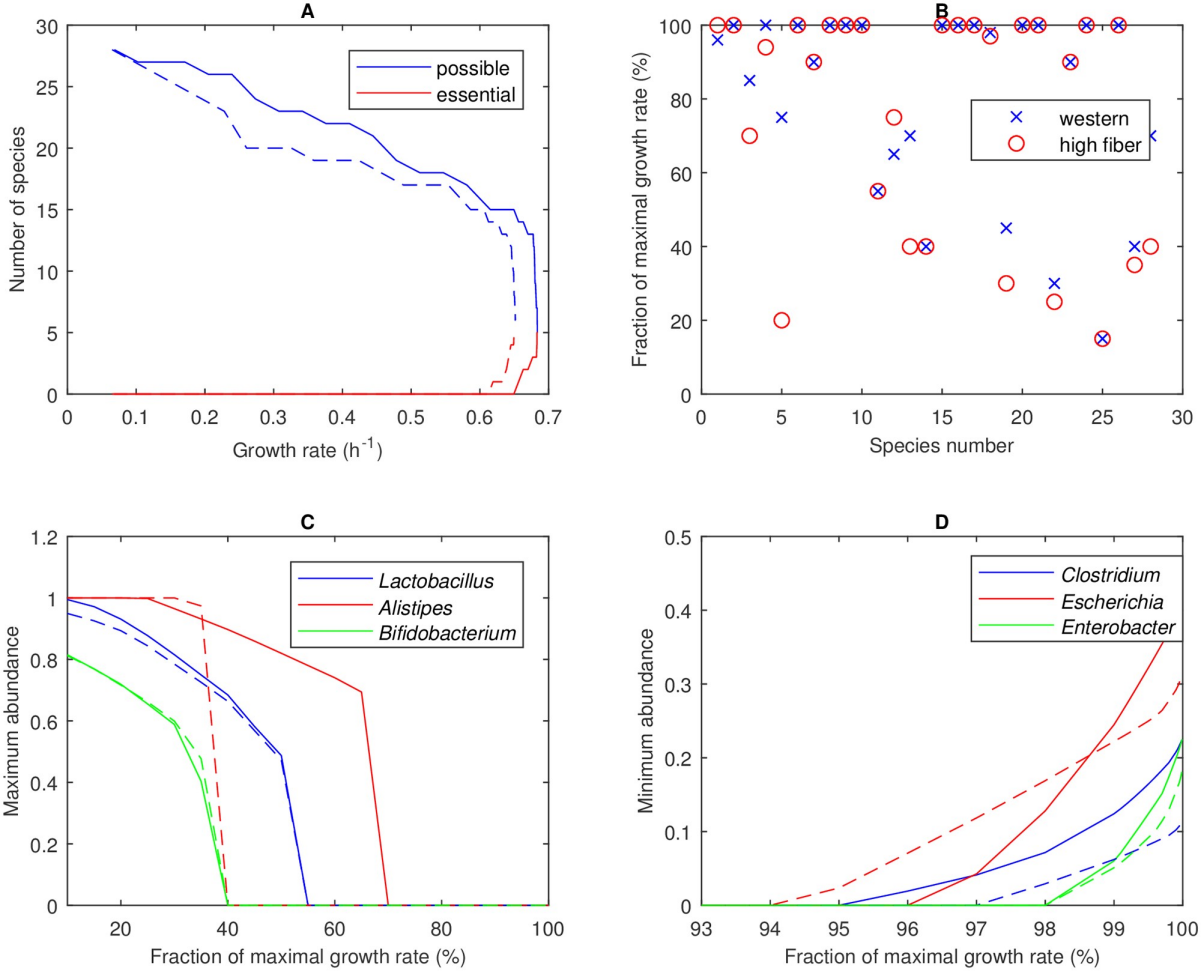
Community FVA performed for the 28-species community with Western (solid lines) and high-fiber (dashed lines) diets. (A) Number of possible species, defined as a species for which its abundance could be maximized to exceed 1%, and essential species, defined as a species for which its abundance could be minimized to be exceed 1%. (B) Community growth rate at which the abundance of each species could no longer exceed 1% when maximized with species numbers corresponding to Table 1. (C) Three representative species that could not coexist below 70% of the maximal growth rate. (D) Three representative species that had to be present in the community at high growth rates.

*In silico* communities optimized for maximal growth exhibited a lack of species diversity and SCFA imbalance characterized by low butyrate levels, both of which are strongly correlated to gut disease [5, 6, 20, 53]. To explore species diversity and SCFA synthesis at suboptimal growth rates, SteadyCom was used to perform community flux variability analysis (FVA) with respect to the species abundances. For growth rates between 10% and 99.99% of the maximal value, the number of species abundances that could be maximized to exceed 1% of the community abundances (possible species) or could be minimized to be exceed 1% of the community abundances (essential species) were determined. Uniqueness of the maximal growth communities was indicated by the convergence of the number of possible and essential species to a single value at the maximal growth rate (Fig 3A). FVA produced unique species abundances for all maximal growth communities S1 Folder. While the Western diet could only support five species at the maximal growth rate, the possible community size increased to 13 species at 99% and to 18 species at 80% of the maximal growth rate. Nine species were predicted to be incapable of coexistence at growth rates greater than 70% of the maximal value (Fig 3B), including saccharolytic *Alistipes* and the common probiotics *Lactobacillus* and *Bifidobacterium* (Fig 3C). No species were essential until 96% of the maximal growth rate and all five species that comprised the optimized community were not essential until 99.92% (Fig 3D). Similar results were obtained with the high-fiber diet. These results suggest that substantially enhanced diversity may be achievable at even marginally suboptimal growth rates.

**Fig 4:**
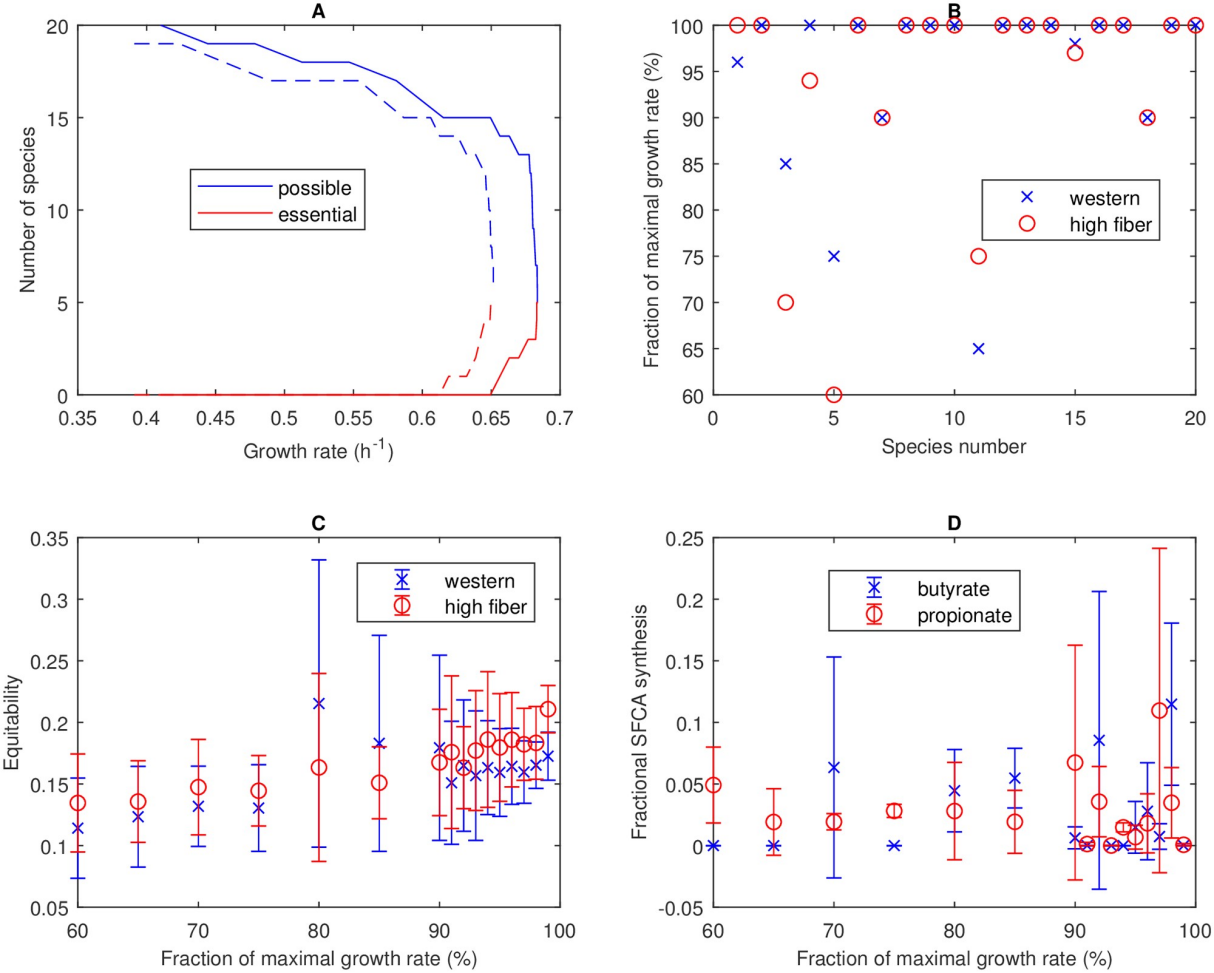
Community FVA performed for the 20-species community with Western and high-fiber diets. (A) Number of possible species and essential species for Western (solid lines) and high-fiber (dashed lines) diets. (B) Growth rate at which the abundance of each species could no longer exceed 1%. (C) Equitability measure *D*_*com*_ calculated from the 40 FVA solutions at each growth rate. Symbols represent the mean and error bars represent the standard deviation. (D) Fractional butyrate and propionate synthesis, defined as the synthesis rate of the SCFA divided by the synthesis rate of all SCFAs (acetate, butyrate and propionate), calculated from the 40 FVA solutions at each growth rate for the Western diet. Symbols represent the mean and error bars represent the standard deviation.

Based on the results in Fig 3B, the 28-species community was reduced to a 20-species community by removing eight species that could only coexist at growth rates less than 70% of the maximal value on both diets (Table 1). These species belonged to the genera *Lactobacillus*, *Alistipes*, *Bifidobacterium*, *Parabacteroides*, *Pseudoflavonifractor*, *Phascolarctobacterium*, *Megasphaera* and *Acidaminococcus*. This reduction in community size allowed a more efficient exploration of species diversity at growth rates above 70% of the maximal value, where the eliminated species were ensured not to coexist. Community FVA performed with the 20-species community suggested that 18 species could coexist at 80% and 15 species could coexist at 95% of the maximal growth rate on the Western diet (Fig 4A). Similar results were obtained for the high-fiber diet. By construction, all species could coexist at 70% of the maximal growth rate for at least one diet (Fig 4B).

We hypothesized that the tradeoff between community growth and species diversity could be described by a Pareto optimal surface [49, 50, 63–65] with increased diversity achieved only at the expense of reduced growth. However, community FVA did not necessarily provide a means to sample this tradeoff surface since diversity is not considered as part of the analysis. To investigate this issue, the 40 FVA solutions (20 solutions each for species abundance minimization and maximization) generated at each growth rate were used to compute the equitability measure *D*_*com*_. The average *D*_*com*_ value for all growth rates and both diets was in the small range 0.11–0.22 (Fig 4C). Of the 2,720 FVA solutions tested, the largest *D*_*com*_ was 0.43 and only nine cases produced *D*_*com*_ > 0.35. In other words, the FVA solutions proved inadequate for generating high diversity, which was not surprising given that FVA solutions were computed by minimizing or maximizing a particular species abundance. This lack of diversity again translated to SCFA imbalance, with the average butyrate and propionate fractions over all 1,360 cases for the Western diet being 2% and 3%, respectively (Fig 4D).

### Modulation of metabolite crossfeeding establishes tradeoff between growth and diversity

**Fig 5:**
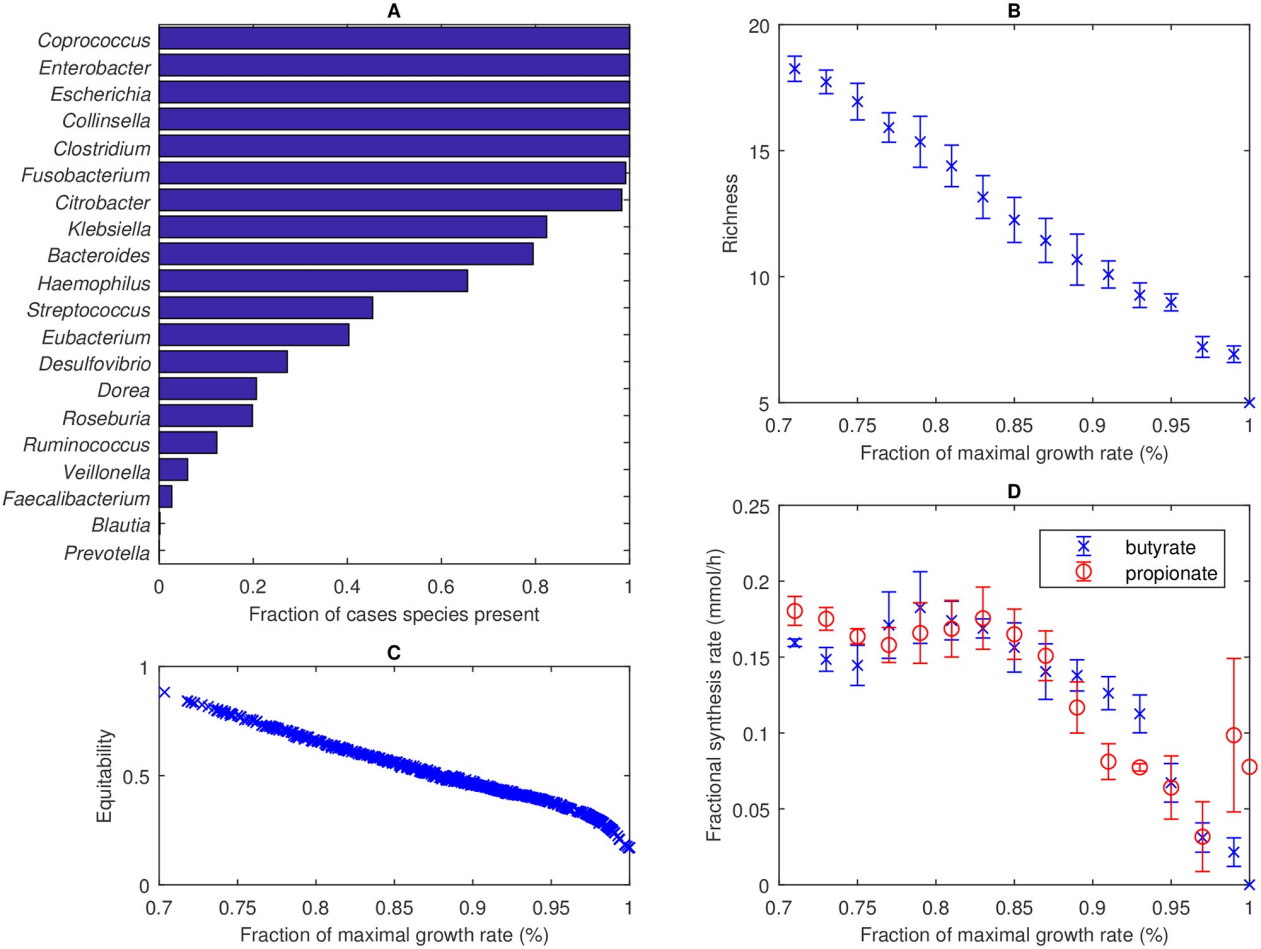
Optimized 20-species communities for the Western diet with species abundance bounds calculated from community FVA solutions. (A) Fraction of the 575 simulation cases for which the species abundance exceeded 1%. (B) Binned community richness, defined as the number of species with abundances greater than 1%. Results of the 575 cases were collected into 15 bins centered at growth rates ranging from 0.71 to 0.99 of the maximal value. The symbols represent the mean and error bars represent the standard deviation in each bin. (C) Equitability measure *D*_*com*_ calculated for all 575 cases. (D) Binned fractional butyrate and propionate synthesis.

While useful for generating bounds on achievable species diversity and SCFA production, community FVA did not provide a direct means to investigate the tradeoff between community growth and diversity. However, we found that this tradeoff could be explored effectively by using FVA solutions at a particular growth rate as inputs to Eq 2 for calculation of an upper bound on each species abundance. When FVA solutions at low growth rates (*e.g.* 60% of maximal) were used for constraint calculation, the resulting solutions tended to have relatively high diversity and low growth. Using 575 simulation cases performed with the 20-species community and the Western diet, the fraction of cases in which the abundance of each species exceeded 1% was calculated as a measure of species fitness over a wide range of growth rates (Fig 5A). The five species that comprised the community at the optimal growth rate were present in all 575 communities. *Citrobacter* and the high butyrate producer *Fusobacterium* were present in over 98% of communities, while *Klebsiella* and the high propionate producer *Bacteroides* were present in at least 80% of communities. By contrast, the high butyrate producer *Faecalibacterium* and and high propionate producer *Veillonella* were present in no more than 6% of communities. Similar results were obtained with the high-fiber diet (S2A Fig).

Results of the 575 Western diet cases were collected into 15 bins in the growth rate space. While the maximal growth solution consisted of only five species with non-zero abundances, the richness of suboptimal solutions was found to routinely exceed ten species (Fig 5B) with a maximum richness of 19 species achieved at 70.3% of the maximal growth rate. The binned richnesses showed small variabilities, partially due to the use of FVA solutions with zero maximum abundance values for some species. Similar richness trends were observed for the high-fiber diet, with 61% and 18% of the 568 simulation cases having richnesses of at least 10 and 15 species, respectively (S2B Fig).

The 575 Western diet cases produced a remarkably simple tradeoff between community growth and species diversity represented by a line (*R*^2^ = 0.996) for growth rates less than 97% of the optimal value (Fig 5C). We hesitate to refer this curve as “Pareto optimal” because our computational procedure does not ensure Pareto optimality of the calculated points. Regardless, this curve clearly showed that species diversity could only be achieved at the expense of community growth and visa versa. A very similar growth-equitability curve was generated with the high-fiber diet (S2C Fig).

Enhanced species diversity at suboptimal growth rates tended to produce more favorable ratios of SCFA net synthesis rates (Fig 5D). For the Western diet, the bin centered at 0.79 produced average butyrate and propionate fractions of 18% and 17%, respectively, which appeared to be more consistent than the maximal growth solution with the 20% values commonly reported for *in vivo* levels of these two SCFAs. The binned SCFA fractions showed small variabilities, consistent with predicted richness variations. Less favorable SCFA synthesis rates were predicted for the high-fiber diet (S2D Fig). While the propionate fractional rate averaged 18% over the range of 70–95% of the maximal growth rate, the butyrate fractional rate never averaged more 12% in any bin and reached a single-case maximum of 13%. Lower butyrate synthesis compared to the Western diet was attributed to reduced participation of the high butyrate producers *Fusobacterium*, *Eubacterium* and *Faecalibacterium* in the simulated communities.

While our method of imposing calculated bounds on the species abundances proved effective for investigating the growth-diversity tradeoff, such a direct mechanism for modulating species abundances is not biologically plausible. Several mechanisms for tuning community composition have been widely studied, including spatial structuring of the participating species in multispecies biofilms [66, 67] and the modulation of metabolite crossfeeding between species [68–70]. Because SteadyCom is based on the assumption of a homogeneous environment, the only mechanism available to tune community composition is modulation of nutrient uptake rates, including the uptake rates of crossfed metabolites. To further investigate how crossfeeding was modulated to achieve high diversity at suboptimal growth rates, we compared the optimal solution for the Western diet to the results in Fig 5 for the simulation cases binned at 79% of the optimal growth rate.

**Fig 6:**
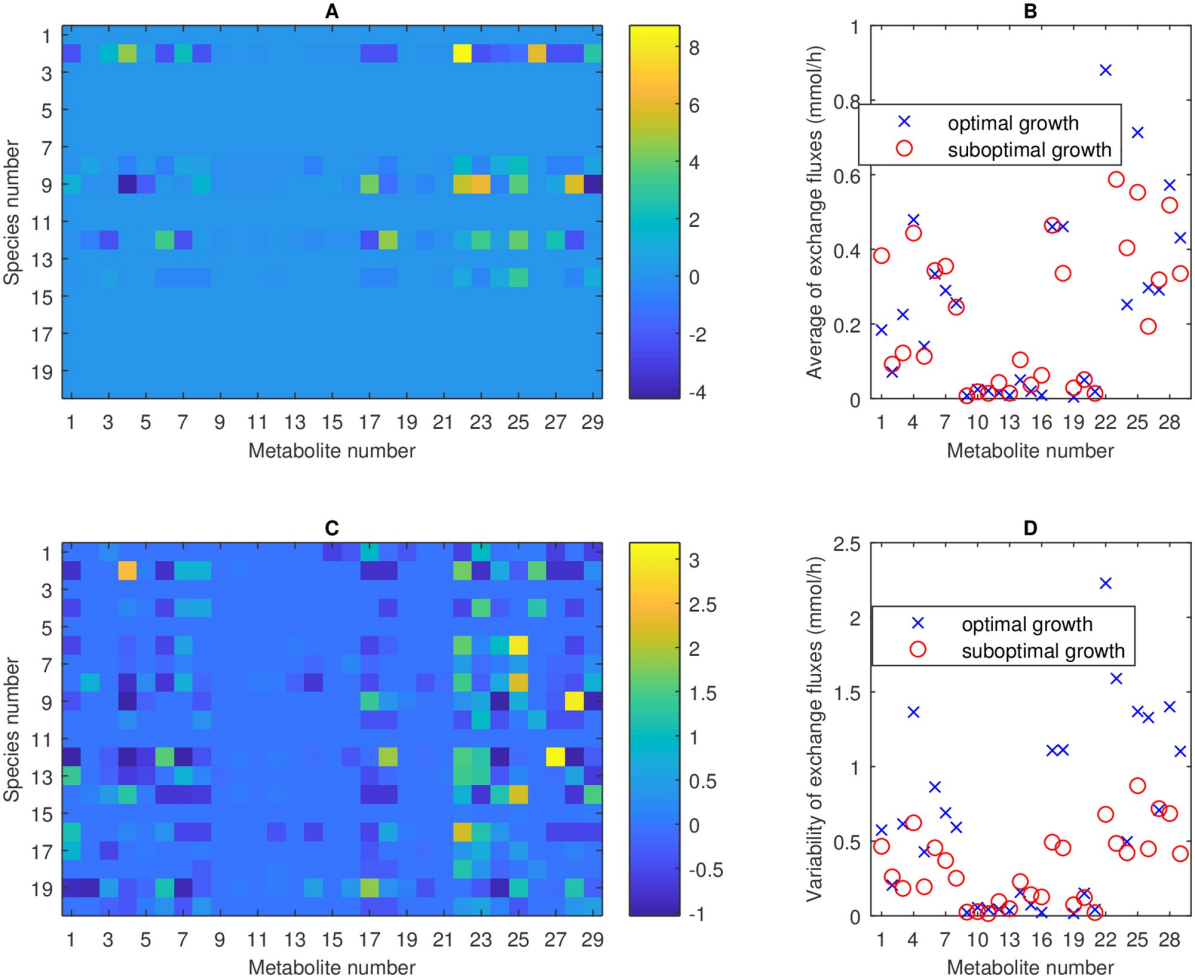
Metabolite crossfeeding for the 20-species community and the Western diet for the maximal growth case and 34 suboptimal growth cases binned around 79% of the maximal growth rate. (A) Maximal growth heatmap of uptake (negative) and secretion (positive) rates in mmol/h for each crossfed metabolite (numbered in S1 Table) and each species (numbered in Table 1). (B) Suboptimal growth heatmap of uptake and secretion rates in mmol/h for each crossfed metabolite and each species averaged across 34 cases. (C) Absolute value of the exchange rate of each crossfed metabolite averaged across the 20 species and the 34 cases (for suboptimal growth). (D) Standard deviation of the exchange rates associated with Fig 6C.

A heatmap of uptake/secretion rates of the 29 crossfed metabolites for each of the 20 species shows that the optimal solution was characterized by relatively high crossfeeding rates between a small number (five) of participating species (Fig 6A). Crossfed metabolites with the largest uptake/secretion rates were aspartate, serine, CO_2_, D-lactate, L-lactate (metabolites 4, 17, 23, 27, 28; see also Fig 2D). Comparable results for suboptimal growth at 79% of the optimal growth rate were generated by averaging the metabolite uptake/secretion rates across the 34 simulation cases within this bin. By contrast to the optimal case, suboptimal growth was characterized by relatively low crossfeeding rates between a large number (15.4 on average) of participating species (Fig 6B).

To better understand the modulation of crossfeeding rates between optimal and suboptimal growth, we averaged the absolute values of the exchange (uptake and secretion) rates of each crossfed metabolite across the 20 species and the 34 simulation cases (for suboptimal growth). While individual crossfed metabolites differed with respect to their average exchange rates, the overall utilization of the 29 metabolites was similar (Fig 6C) with the average exchange rate across all metabolites being 0.25 mmol/h for maximal growth and 0.24 mmol/h for the suboptimal growth cases. By contrast, the standard deviation of the exchange rate across all metabolites was 0.64 mmol/h for maximal growth and 0.32 mmol/h for the suboptimal growth cases. Similar results were obtained for the high-fiber diet (S3 Fig). This analysis reinforced a central theme of this *in silico* study: optimal growth resulted in large metabolite exchange rates between few species while suboptimal growth was characterized by reduced metabolite exchange rates between many species.

## Discussion

Metabolic modeling of the human gut microbiota has emerged as an important *in silico* tool for investigating community growth and composition as well as species interactions. While yielding useful insights into community behavior, previous gut microbiota models [38, 71–75] have been limited with respect to the number of species included and the metabolic interactions allowed. To our knowledge, the gut community models developed in this study represent the most complete descriptions to date both with respect to the number of species (*e.g.* 28 bacteria) and model size (*e.g.* 26,867 metabolites, 35,031 reactions). Our ability to generate and solve such large community models is directly attributable to the availability of gut bacteria reconstructions in the Virtual Metabolic Human database (https://vmh.uni.lu) [44] and the computational efficiency of the SteadyCom method [38]. The model database allowed the species to be chosen based on the most abundant genera in the gut [19] rather than by the availability of curated reconstructions, which remains limited to a few dozen species. The tradeoff for this unprecedented diversity of modeled species was that the reconstructions were only semi-curated and could not be expected to have the fidelity of fully curated models. Given the qualitative nature of our study focused on examining community growth and diversity, this limitation was deemed acceptable. Rather than requiring *a priori* specification of particular interactions between species, SteadyCom allowed arbitrary crossfeeding of secreted metabolites between all species. We limited crossfeeding to the 21 amino acids and 8 common byproducts, yielding 21,924 possible crossfeeding relationships for the 28-species community.

The 28 species included in the first gut community offered a wide diversity of metabolic capabilities, including the synthesis of short-chain fatty acids (SCFAs) used by host colonocytes as a primary energy source. As observed in healthy gut communities [5, 6, 76], SCFA synthesis was diversified across the community, with 24 species, 7 species and 13 species secreting acetate, butyrate and propionate, respectively. However, the maximal growth communities determined with SteadyCom consisted of only five species for the Western diet and six species for the high-fiber diet. The optimized communities were enriched in genera known to be overrepresented in inflammatory bowel disease, namely *Escherichia* [60], *Enterobacter* [61] and *Citrobacter* [62]. The communities also exhibited large imbalances in SCFA production, with over 90% of SCFA synthesis yielding acetate and no butyrate secreted, another hallmark of IBD [20–22]. Our simulation results suggest that maximal community growth unchecked by the host may evolve to disease states such as IBD.

We used SteadyCom to perform community flux variability analysis (FVA) as a means to determine limits on achievable species diversity and SCFA synthesis. FVA suggested that suboptimal community growth rates offered the potential for substantially enhanced diversity with 20 of the 28 species capable of coexisting at 70% of the optimal growth rate for at least one diet. Based on these results, we generated a reduced 20-species community by eliminating the eight species capable of coexisting only at growth rates less than 70% of the maximal values. The eight species eliminated represented several genera known to be beneficial for gut health, most notably *Lactobacillus* [77], *Alistipes* and [78], *Bifidobacterium* [79]. Due to their low predicted growth rates compared to other community members, these species may need to establish favorable metabolic niches along the intestine to robustly coexist [80–82]. While outside the scope this study due to the homogeneous assumption underlying SteadyCom, the effect of such spatial gradients would be interesting topic for future research.

FVA performed with the reduced 20-species community further demonstrated the potential for achieving high species richness (defined as the number of species with abundances of at least 1%) and equitability (see Eq 1) as well as balanced SCFA production at suboptimal growth rates. We developed a simple randomized method for using the FVA results to constrain species abundances in SteadyCom to achieve suboptimal growth rates and sample the growth-diversity tradeoff surface. A remarkably simple linear relationship between the community growth rate and species equitability was predicted, with high levels of diversity (richness ≥ 12 species, equitability ≥ 0.55) achievable at growth rates below 85% of the maximal value. Increased species diversity resulted in more balanced SCFA synthesis, with butyrate comprising 14–18% of total production. These predictions are consistent with known characteristics of healthy gut communities, suggesting that simulated suboptimal growth represents a “healthy” state and simulated maximal growth represents a “dysbiosis” state such as IBD.

We further analyzed our suboptimal growth solutions to determine how SteadyCom achieved enhanced diversity and how the host might promote diversity *in vivo*. In addition to modulating the uptake of available nutrient across species, SteadyCom tuned the secretion/uptake rates of crossfed amino acids and metabolic byproducts to enhance the growth rates of otherwise slower growing species. Compared to maximal growth, suboptimal solutions were characterized by lower secretion and uptake rates of crossfed metabolites between a larger number of species. These results suggest modulation of crossfeeding relationships is one possible mechanism available to the host for promoting diversity at the expense of growth. From a theoretical perspective, host-microbiota metabolic interactions might be viewed as a type of bilevel optimization problem with the microbiota attempting to achieve maximal community growth and the host modulating the gut environment to maximize species diversity. Gut diseases such as IBD might result from the host “losing the battle” due to inflexibilities resulting from poor diet and/or sudden loss of diversity due to antibiotic treatment. While a few metabolic modeling methods that address diversity have been proposed [83–85], additional modeling tools that directly address the growth-diversity tradeoff in microbial communities are needed.

As has been reported in numerous *in vivo* studies [86–89], we expected the *in silico* high-fiber diet to promote species diversity and enhance butyrate synthesis as compared to the Western diet. Instead our model predicted that diet had little effect on the growth-diversity tradeoff and high fiber actually resulted in reduced butyrate levels. These discrepancies could reflect insufficient numbers of fiber-degrading and butyrate-producing species in our simulated communities. Indeed only four members (*Bacteroides*, *Prevotella*, *Alistipes*, *Desulfovibrio*) of the 28-species community exhibited faster single-species growth rates on the high-fiber diet, and two of these species were removed to generate the 20-species community. The communities contained only three major butyrate producers, with one species (*Faecalibacterium*) being among the least competitive members of the 20-species community. The computational efficiency of SteadyCom allows the construction of larger community models with more representation of fiber-degrading and butyrate-producing species.

## Supporting information

S1 Fig Optimized 28-species communities for the *in silico* Western diet with all ATP maintenance values set to 10 mmol/gDW/h or tuned as possible to achieve equal single-species growth rates of 0.2 h^−1^. (A) Growth rate (h^−1^), non-zero species abundances and equitability measure *D*_*com*_. (B) Single-species growth rates (h^−1^) with species numbers corresponding to Table 1. (C) Net synthesis rates of major metabolic byproducts including the three SCFAs acetate, butyrate and propionate. The byproducts ethanol, D-lactate, L-lactate and succinate are not shown because their net secretion rates were zero. (D) Uptake and secretion fluxes of the five metabolites most significantly crossfed between the participating species.
(TIF)

S2 Fig Optimized 20-species communities for the high-fiber diet with species abundance bounds calculated from community FVA solutions. (A) Fraction of the 568 simulation cases for which the species abundance exceeded 1%. (B) Binned community richness, defined as the number of species with abundances greater than 1%. Results of the 568 cases were collected into 15 bins centered at growth rates ranging from 0.71 to 0.99 of the maximal value. The symbols represent the mean and error bars represent the standard deviation in each bin. (C) Equitability measure *D*_*com*_ calculated for all 568 cases. (D) Binned fractional butyrate and propionate synthesis.
(TIF)

S3 Fig Metabolite crossfeeding for the 20-species community and the high-fiber diet for the maximal growth case and 36 suboptimal growth cases binned around 79% of the maximal growth rate. (A) Maximal growth heatmap of uptake (negative) and secretion (positive) rates in mmol/h for each crossfed metabolite (numbered in S1 Table) and each species (numbered in Table 1). (B) Suboptimal growth heatmap of uptake and secretion rates in mmol/h for each crossfed metabolite and each species averaged across 36 cases. (C) Absolute value of the exchange rate of each crossfed metabolite averaged across the 20 species and the 36 cases (for suboptimal growth). (D) Standard deviation of the exchange rates associated with S3 FigC.
(TIF)

S1 Table Crossfed amino acids and metabolic byproducts. (PDF)

S1 File Uptake constraints. Community uptake constraints for the four models (20 and 28 species; Western and high-fiber diets).
(XLSX)

S1 Folder Matlab codes. Matlab codes and data files used for generating results.
(ZIP)
