# Supplementary figures and images for: Suboptimal community growth mediated through metabolite crossfeeding promotes species diversity in the gut microbiota
Source: PLoS Comput Biol. 2018 Oct 30;14(10):e1006558. doi: 10.1371/journal.pcbi.1006558 (PMC6226200; doi:10.1371/journal.pcbi.1006558)

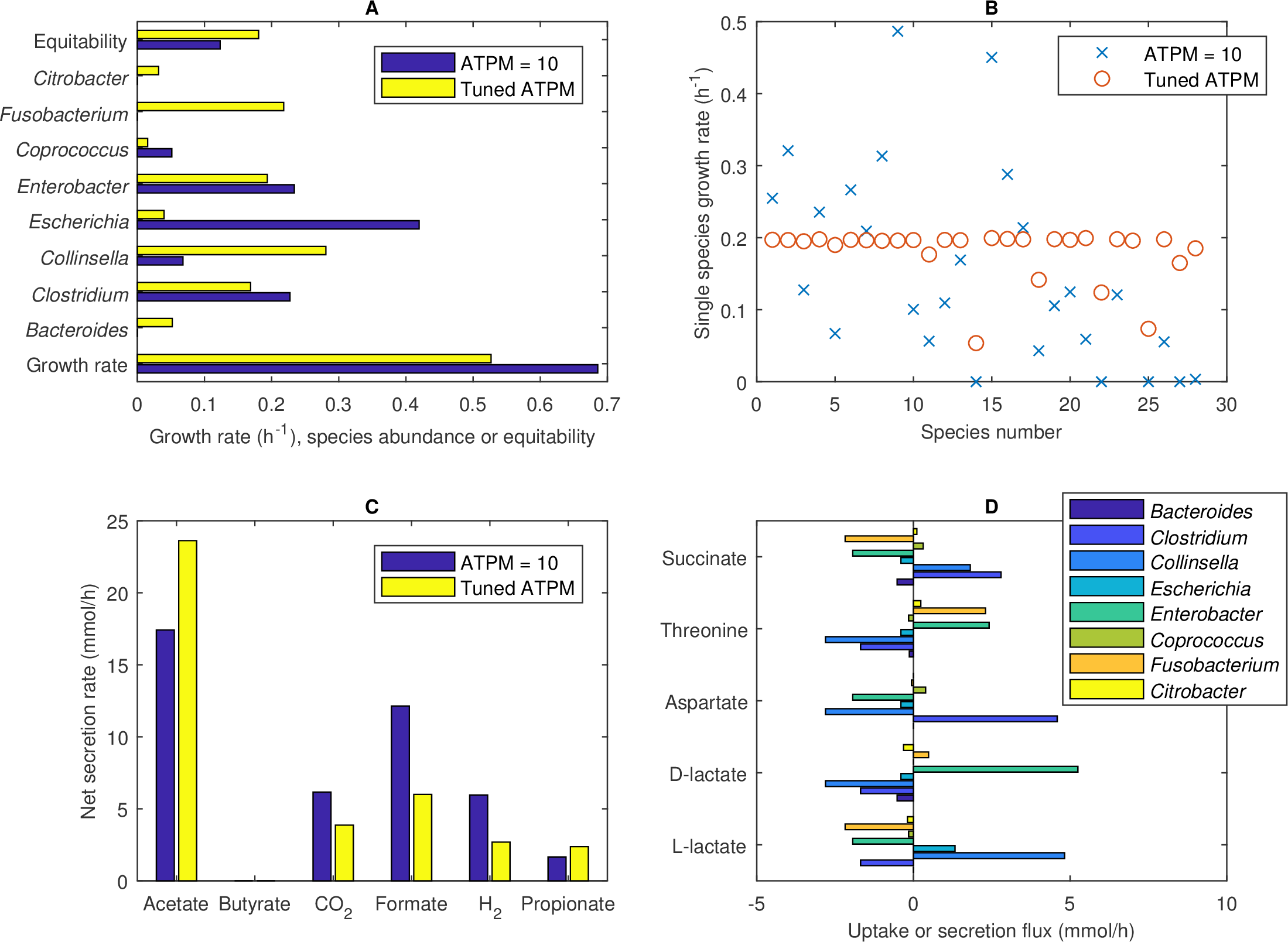

Supplement: S1 Fig — (A) Growth rate (h−1), non-zero species abundances and equitability measure Dcom. (B) Single-species growth rates (h−1) with species numbers corresponding to Table 1. (C) Net synthesis rates of major metabolic byproducts including the three SCFAs acetate, butyrate and propionate. The byproducts ethanol, D-lactate, L-lactate and succinate are not shown because their net secretion rates were zero. (D) Uptake and secretion fluxes of the five metabolites most significantly crossfed between the participating species. (TIF) [file pcbi.1006558.s001.tif]

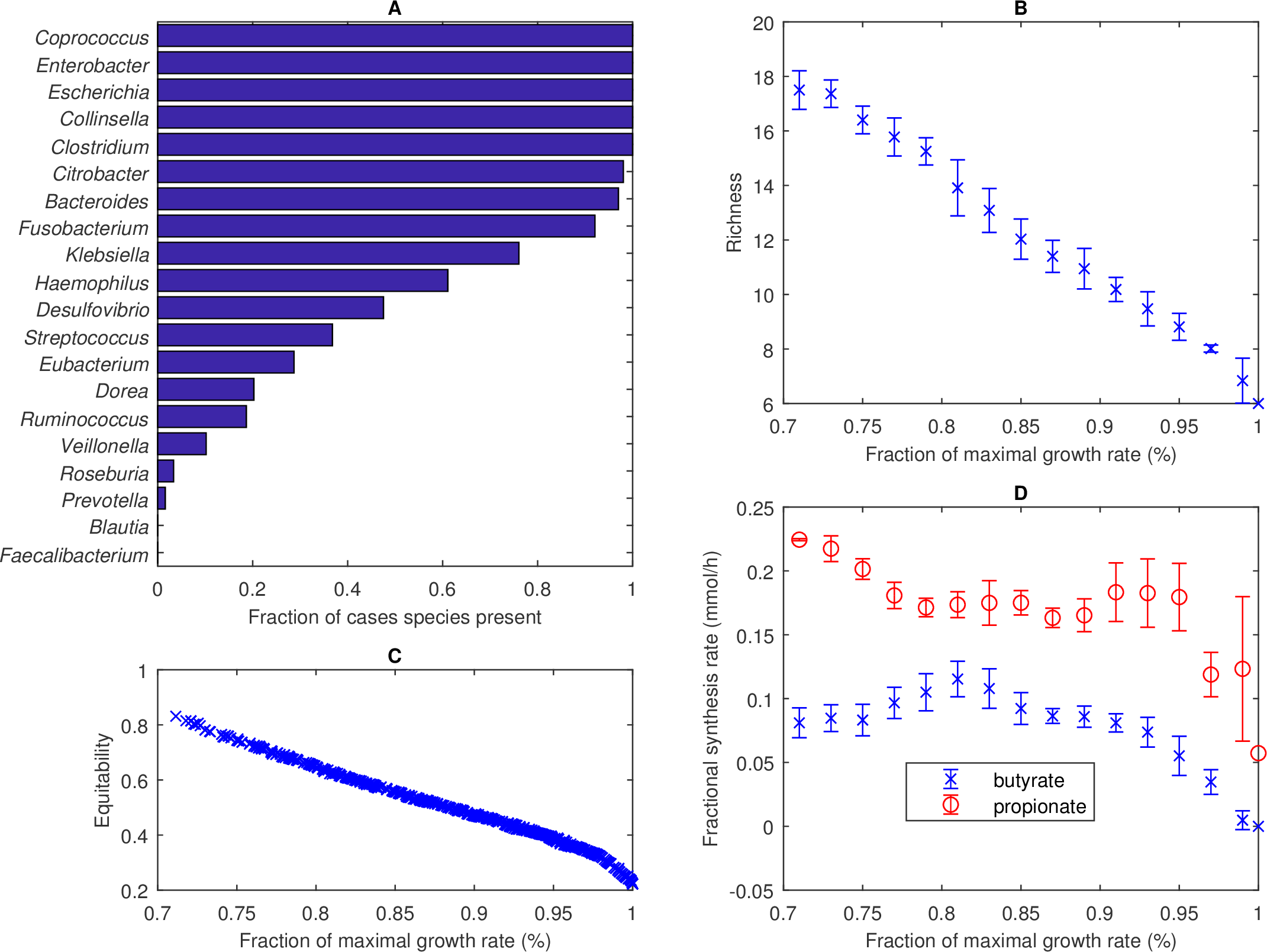

Supplement: S2 Fig — (A) Fraction of the 568 simulation cases for which the species abundance exceeded 1%. (B) Binned community richness, defined as the number of species with abundances greater than 1%. Results of the 568 cases were collected into 15 bins centered at growth rates ranging from 0.71 to 0.99 of the maximal value. The symbols represent the mean and error bars represent the standard deviation in each bin. (C) Equitability measure Dcom calculated for all 568 cases. (D) Binned fractional butyrate and propionate synthesis. (TIF) [file pcbi.1006558.s002.tif]

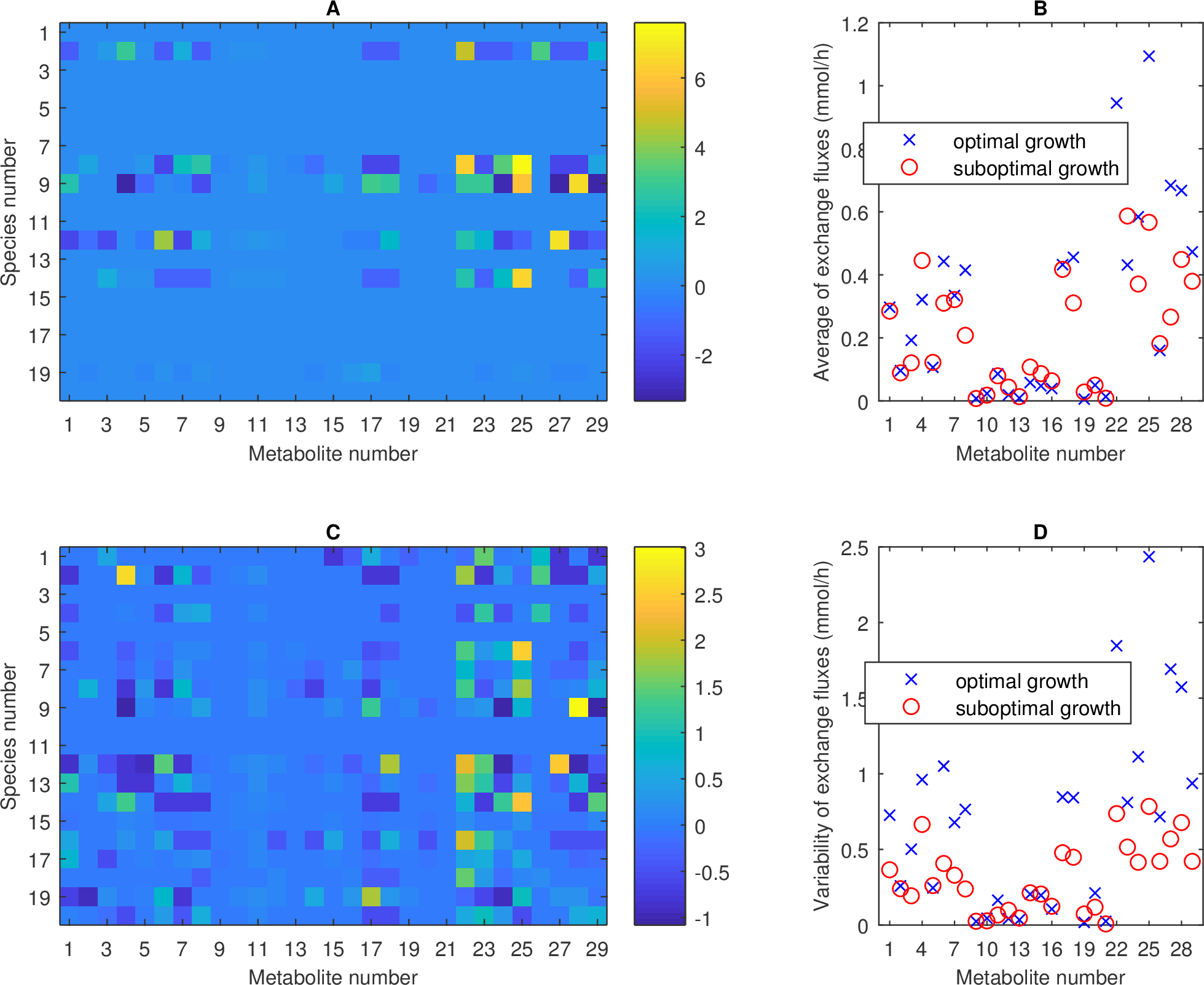

Supplement: S3 Fig — (A) Maximal growth heatmap of uptake (negative) and secretion (positive) rates in mmol/h for each crossfed metabolite (numbered in S1 Table) and each species (numbered in Table 1). (B) Suboptimal growth heatmap of uptake and secretion rates in mmol/h for each crossfed metabolite and each species averaged across 36 cases. (C) Absolute value of the exchange rate of each crossfed metabolite averaged across the 20 species and the 36 cases (for suboptimal growth). (D) Standard deviation of the exchange rates associated with S3 FigC. (TIF) [file pcbi.1006558.s003.tif]
