# Supplementary material for: Suboptimal community growth mediated through metabolite crossfeeding promotes species diversity in the gut microbiota
Source: PLoS Comput Biol. 2018 Oct 30;14(10):e1006558. doi: 10.1371/journal.pcbi.1006558 (PMC6226200; doi:10.1371/journal.pcbi.1006558)
Supplement: S1 Table — (PDF) [file pcbi.1006558.s004.pdf]

Crossfed amino acids and metabolic byproducts.

Number	Metabolite	Number	Metabolite
1	L-alanine	17	L-serine
2	L-arginine	18	L-threonine
3	L-asparagine	19	L-tryptophan
4	L-aspartate	20	L-tyrosine
5	L-cysteine	21	L-valine
6	L-glutamine	22	acetate
7	L-glutamate	23	butyrate*
8	glycine	24	CO_2
9	L-histidine	25	ethanol
10	L-isoleucine	26	formate
11	L-leucine	27	H_2
12	L-lysine	28	D-lactate
13	L-methionine	29	L-Lactate
14	ornithine	30	propionate*
15	L-phenylalanine	31	succinate
16	L-proline		

SCFAs not allowed to be crossfed

S1 Table.
